# Noninvasive Suspicious Liquid Detection Using Wireless Signals

**DOI:** 10.3390/s19194086

**Published:** 2019-09-21

**Authors:** Jiewen Deng, Wanrong Sun, Lei Guan, Nan Zhao, Muhammad Bilal Khan, Aifeng Ren, Jianxun Zhao, Xiaodong Yang, Qammer H. Abbasi

**Affiliations:** 1 School of Electronic Engineering, Xidian University, Xi’an 710071, China; djw15529256085@163.com (J.D.); sunwanrong@xidian.edu.cn (W.S.); nan_zhao_@hotmail.com (N.Z.); engrmbkhan1986@gmail.com (M.B.K.); afren@mail.xidian.edu.cn (A.R.); jxzhao@xidian.edu.cn (J.Z.); 2 School of Life Sciences and Technology, Xidian University, Xi’an 710126, China; 15926395470@163.com; 3 School of Engineering, University of Glasgow, Glasgow G12 8QQ, UK; Qammer.Abbasi@glasgow.ac.uk

**Keywords:** 5G, liquid detection, radio propagation, dielectric constant, WCI

## Abstract

Conventional liquid detection instruments are very expensive and not conducive to large-scale deployment. In this work, we propose a method for detecting and identifying suspicious liquids based on the dielectric constant by utilizing the radio signals at a 5G frequency band. There are three major experiments: first, we use wireless channel information (WCI) to distinguish between suspicious and nonsuspicious liquids; then we identify the type of suspicious liquids; and finally, we distinguish the different concentrations of alcohol. The K-Nearest Neighbor (KNN) algorithm is used to classify the amplitude information extracted from the WCI matrix to detect and identify liquids, which is suitable for multimodal problems and easy to implement without training. The experimental result analysis showed that our method could detect more than 98% of the suspicious liquids, identify more than 97% of the suspicious liquid types, and distinguish up to 94% of the different concentrations of alcohol.

## 1. Introduction

The illegal carrying and transportation of flammable and explosive liquids seriously affects public safety. Flammable and explosive liquids such as gasoline and alcohol are also commonly used in various terrorist activities. Therefore, the safety inspection of flammable and explosive liquids is of great significance for ensuring public safety. It has broad application prospects in the fields of public security, civil aviation, and customs. Liquids cannot be identified by the naked eye, and it is difficult to carry out dangerous liquid inspection in crowded places, which is a great challenge for security personnel. Moreover, there are still some places where the “taste liquid” method is used to determine whether the liquid is safe [1]. Forbid passengers to carry large amounts of liquid has become the main method to prevent terrorist attacks. For example, international civil aviation regulations prohibit carrying more than 100 mL of liquid, and trains and high-speed trains prohibit carrying flammable liquids and more than 120 mL of compressed spray. The traditional detection of suspicious items is either manual inspection (such as setting checkpoints at each entrance) or special equipment (such as surveillance cameras, X-ray machines, and ultra-wideband scanners), which is costly, expensive to deploy, and difficult to implement on a large scale [2]. It is necessary to introduce a new suspicious liquid detection scheme which is more economical and covers a wider range.

There are several mature liquid detection technologies. A traditional approache, the Raman spectrum analysis, uses the molecular structure to identify liquids according to their scattering spectroscopy. Raman spectroscopy has many unique advantages, such as wide detection range, sharp spectral peaks, and high resolution [3]. That is, when monochromatic light radiates on an object, the molecules of the substance will scatter, and the spectrum reflected by different substances will be different. Moreover, the method, based on X-ray image technology, is used to identify liquid substances, and can obtain the atomic number of the liquid through its X-ray [4]. However, this method has a certain error rate and the equipment is very expensive. Another method is to detect liquids based on the different absorption and attenuation characteristics of different substances via electromagnetic waves [5,6]. Microwave detection can achieve noncontact detection with a certain distance between the detected objects, and has high detection sensitivity. However, these approaches rely on expensive and specialized equipment, which does not facilitate wide deployment in practice.

Recently, Radio Frequency (RF) based sensing has drawn considerable attention. A couple of studies have explored the feasibility of using RF signals for remote monitoring and controlling during infusion. For instance, a wireless intelligent monitoring system, based on wireless communication and network technology, is put forward, which can monitor drip speed in real time and automatically alarm in abnormal conditions [7]. The ZigBee wireless sensor is used to detect the velocity of liquid droplets [8]. While these approaches mainly focus on exploiting the differences of wireless signal measurements to sense physical morphological changes of liquids, using fine-grained wireless channel information (WCI) to identify types of liquid remains an option.

5G communication technology is a data and information transmission technology developed by technicians based on 4G technology. Its advantages include sound transmission performance, fast transmission speed, high utilization rate of resources, and wide range of coverage. As such, it is favored in modern data and information transmission [9]. Based on ubiquitous wireless signals, the wireless sensing system will provide a variety of high precision, high reliability, high security, and convenient application services, among which the human behavior identification technology is at the core for public use [10]. As the information of the physical layer, the fine-grained WCI contains a lot of channel information which is invisible to the Medium Access Control (MAC) layer. The WCI can measure the frequency response of multiple subcarriers at the same time from a single packet, rather than the overall amplitude response superimposed by all subcarriers. Information about the WCI and frequency-selective channels is described [11].

In the field of wireless sensing, WCI has gradually become a popular area of research [12], such as indoor positioning [13,14,15], respiration detection [16,17,18], and behavior recognition [19,20]. Zhou et al. proposed using the WCI to detect the presence of people in the environment [21]. Although wireless signals have good applications in indoor positioning and fall detection, there are still relatively few studies on detecting the types and concentrations of liquids by using them. Liquid interferes with the path of radio signals. Different liquids have different degrees of interference to the radio signal propagation path due to their differences in dielectric constants, which leads to different changes in the WCI. These changes can be effectively observed at the signal receiver, so as to realize the detection and identification of various liquids. Table 1 shows the relative dielectric constants of common objects.

Following this introduction, the paper is organized into four sections. Section 2 describes the preparatory work. Section 3 details the band selection and method design. Results are analyzed in Section 4 and conclusions are drawn in Section 5.

## 2. Preparatory Work

### 2.1. Data Acquisition

The WCI represents the channel state of a communication link on Orthogonal Frequency Division Multiplexing (OFDM) technology. The WCI describes how links are transmitted from a transmitter to a receiver. It also combines the influence of scattering, fading, power attenuation, and other factors. The WCI reflects the performance of a link and the interference caused by other factors to a great extent [22]. The WCI is composed of an OFDM matrix containing 30 subcarriers. Data can be transmitted simultaneously on multiple subcarriers, greatly improving the efficiency and accuracy of the system [23]. The frequency domain of the wireless channel can be expressed as:

(1) $$\vec{Y}=\;H\;\cdot \;\vec{X}+\vec{N},$$

where $\vec{X}$ and $\vec{Y}$ are the transmitted and received vectors while $\vec{N}$ is Gaussian noise (AWGN) vector and *H* represents the frequency response of the channel.

The receivers receive packets for each subcarrier from a channel. The packet carrying the original amplitude and random phase information complex frequency domain can be expressed as:

(2) $$H(n)\;=\;|\;H(n)|\;e^{∠H\left(n\right)}.$$

In Equation (2), H(n) is the data for subcarrier number n, where n ∈ [1 to 30]. |H(n)| is the raw amplitude information and ∠H(n) denotes the random phase data.

### 2.2. Data Preprocessing

Our system uses a Hampel filter to eliminate the singular value in the data and construct a scale sequence with the median. Assuming that the median of the sequence is Z,

(3) $$\{d(k)\}\;=\;\{|x_{0}(k)-Z|,\;\ldots ,\;|x_{m-1}(k)-Z|\}.$$

The deviation scale of each data from the reference value is given. Suppose the median of {d(k)} is D. The median has an absolute deviation of:

MAD = 1.4826 × D.(4)

MAD can replace the standard deviation σ. The Hampel filter uses m data in a mobile window to determine the validity of current data. If the data is valid, process it; otherwise, replace it with the median. The Hampel filter can protect the detailed information while filtering the singular value.

### 2.3. Classification Method

We used the K-Nearest Neighbor (KNN) algorithm to classify the amplitude information extracted from the WCI matrix to detect liquids. The KNN algorithm mainly relies on the surrounding adjacent samples to determine the category. If the k closest neighbors of a sample belong to a certain category in the feature space, the sample also belongs to the same category. In the KNN algorithm, the selected neighbors are all objects that have been correctly classified. Therefore, the KNN method is more suitable than other algorithms for the sample sets to be divided with a lot of crossover or overlap of the class domain.

The KNN algorithm takes the distance between objects as the nonsimilarity index to avoid the matching problem between objects. Using different distance calculation methods, there may be significant differences between the “neighbors” identified. We used the Euclidean distance method:

(5) $$d\;(x,\;y)\;=\;\sqrt{\sum_{k=1}^{n}{\left(x^{k}-y^{k}\right)}^{2}},$$

In the KNN algorithm, the choice of the K value will have a significant impact on the classification results. Generally, we can take a relatively small value of K, and cross validation is used to select the best value of K. Usually, K is an integer less than 20.

## 3. Method Design

### 3.1. Band Selection

The C-band is a frequency band from 4.0 to 8.0 GHz, which is used as the frequency band for downlink transmission of communication satellite signals. In the application of satellite television broadcasting and various small satellite ground stations, the band was first adopted and has been widely used. The Ministry of Industry and Information Technology (MITT) has announced the frequency band division of China’s fifth generation of mobile communications in its latest official document [24]. The 4.8–5 GHz frequency band in China’s 5G is located in the C-band, which is an important part of 5G communication in China.

The S-band refers to the electromagnetic wave band with a frequency range of 2–4 GHz, which is mainly used in relaying, satellite communication, radar, and so on. Now widely used in Bluetooth, ZigBee, wireless routing, and wireless mouse devices also use S-band electromagnetic waves.

Many researches have indicated that the longer the wavelength of the electromagnetic wave, the stronger its ability to diffract. For example, radio waves can be transmitted around tall buildings, and red light can travel far in fog to remind drivers, which is more effective than green light and yellow light. The shorter the wavelength of the electromagnetic wave, the greater the energy of the wave, and the stronger the penetrating capacity. As such, X-ray can penetrate through skin and bones, ultraviolet rays can kill bacteria, and strong ultraviolet rays can cause skin cancer.

Therefore, we selected electromagnetic waves of two frequency bands to study the influence of electromagnetic waves of different bands on liquid detection. We chose a 2.4 GHz signal located in the S-Band and a 4.8 GHz signal located in the C-band (5G frequency band) for comparison.

### 3.2. Method Design

**Figure 1:**
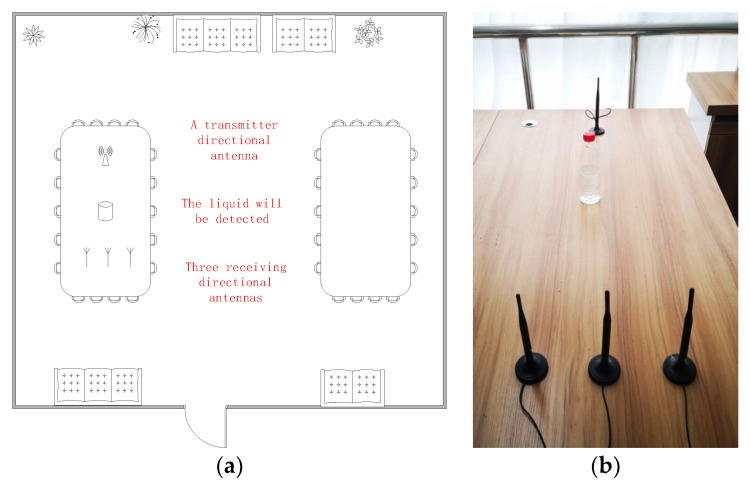
(**a**) Experimental scenario; (**b**) The actual scene.

The experimental scenario used to facilitate the detection and identification of suspicious liquids is shown in Figure 1. Our experiment was conducted in a conference room and used two sets of equipment placed on a desk to collect data. The transmitter and receiver were one meter apart. The liquid to be detected was placed statically between the transmitter and receiver at the same height. One set of equipment worked at 2.4 GHz, and the transmission and reception of signals were completed by the wireless network adapter of the computer and three omnidirectional antennas. The bandwidth was 20 MHz, and the output power of the transmitter was set at −5 dBm. The other worked at 4.8 GHz, which is consistent with the 5G standard in China, with the RF signal generator as the transmitter and spectrum analyzer as the receiver. The bandwidth was 100 MHz, and the output power of the transmitter was set at −5 dBm. The receiver collected the RF signal at the frequency of 4.8 GHz and 2.4 GHz, corresponding to the C-band (5G) and the S-band, respectively.

**Figure 2:**
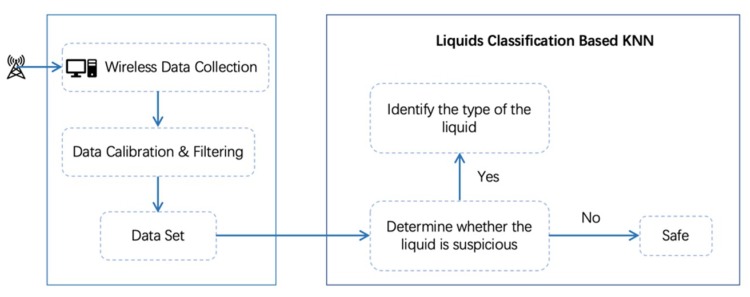
Method flow chart. KNN, K-Nearest Neighbor.

To facilitate the liquid detection and identification leveraging Wi-Fi signal, we exploited wireless channel information (WCI), the fine-grained description of the wireless channel, to capture the minute differences of the channel state change introduced by different liquids. Our method employed the amplitude information of WCI. First, we extracted the WSI from a pair of transmitters and receivers. Second, we preprocessed the data to remove the environmental noise and eliminate the singular value. Then the data were feature selected by a Principal Components Analysis (PCA) algorithm and classified by a K-Nearest Neighbor (KNN) algorithm. The flow chart of the method is shown in Figure 2.

Using this method, we carried out three experiments. Experiment 1 was to distinguish between suspicious and nonsuspicious liquids, Experiment 2 was to identify the type of suspicious liquids, and Experiment 3 was to distinguish the three different concentrations of alcohol.

## 4. Evaluation and Analyses

**Figure 3:**
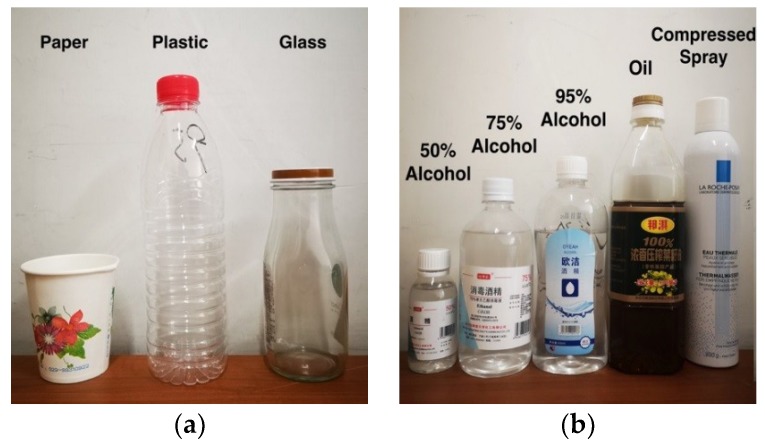
(**a**) The selection of containers; (**b**) Suspicious liquids selected from the experiment.

In this work, containers made of three common materials were selected. Figure 3a shows our selection of containers, from left to right a paper cup, a plastic bottle, and a glass bottle. We used 50% alcohol, 75% alcohol, 95% alcohol, oil, and a compressed spray as the representative of suspicious liquid. We chose water as the representative of nonsuspicious liquid. Figure 3b shows the suspicious liquids selected for the experiment.

### 4.1. Detection of Suspicious and Nonsuspicious Liquids

In this section, we first analyze the detection of suspicious and nonsuspicious liquids.

**Figure 4:**
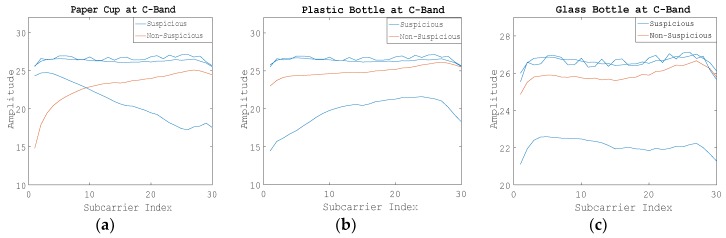
Amplitude information of 30 subcarriers of Step 1 at the C-band. (**a**) Using the paper cup; (**b**) Using the plastic bottle; (**c**) Using the glass bottle.

Figure 4 shows the raw WCI amplitude information on 30 subcarriers when using C-band electromagnetic wave signals. We can see the difference of amplitude change between suspicious liquids and nonsuspicious liquid intuitively.

**Figure 5:**
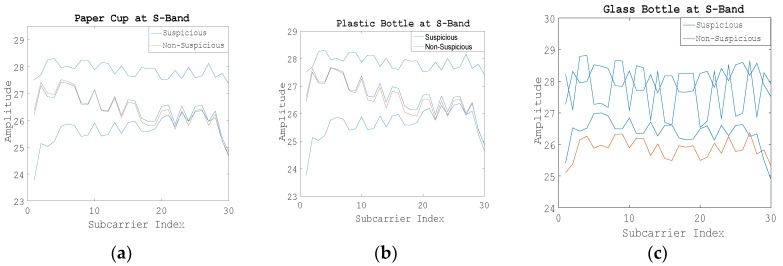
Amplitude information of 30 subcarriers of Step 1 at the S-band. (**a**) Using the paper cup; (**b**) Using the plastic bottle; (**c**) Using the glass bottle.

The conclusion of the S-band was the same, as shown in Figure 5.

**Figure 6:**
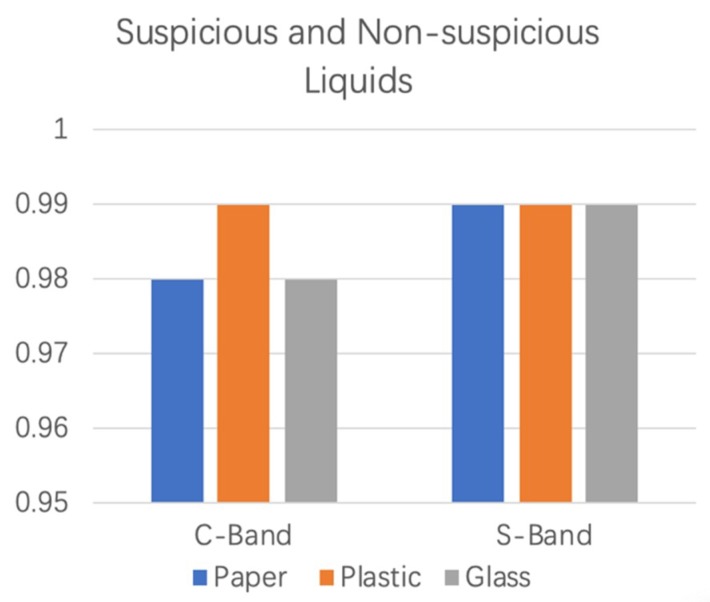
The KNN algorithm Classification results of Step 1 at the C-band and the S-band.

The KNN classification algorithm was used to further detect suspicious and nonsuspicious liquids. The classification results of the KNN algorithm for two bands of data are shown in Figure 6. Blue, orange, and grey represent the paper cup, the plastic bottle, and the glass bottle, respectively. In the C-band environment, the detection accuracy of Step 1 was over 98%, and that for S-band was 99%.

### 4.2. Identification of Suspicious Liquids

In this section, we will analyze the identification of suspected liquids.

**Figure 7:**
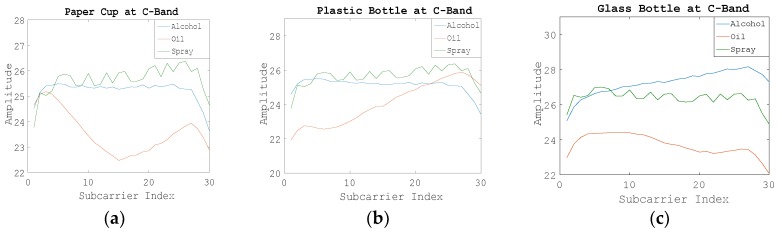
Amplitude information of 30 subcarriers of Step 2 at the C-band. (**a**) Using the paper cup; (**b**) Using the plastic bottle; (**c**) Using the glass bottle.

Figure 7 shows the WCI amplitude information of 30 subcarriers. Blue, orange, and green represent alcohol, oil, and compressed spray, respectively. It can be seen that in the C-band environment, the WCI amplitude ranges of alcohol and oil were not very different, but the amplitude fluctuation trends were obviously different, and the amplitude ranges of these two categories were greatly different from those of oil.

**Figure 8:**
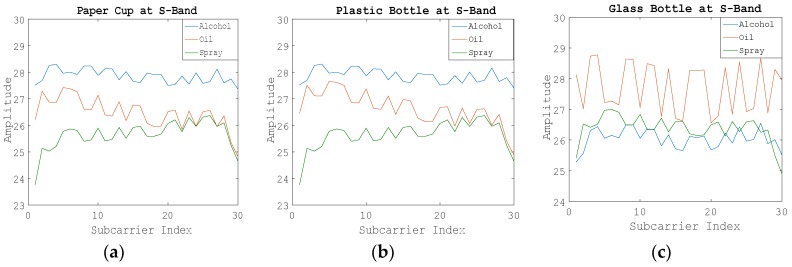
Amplitude information of 30 subcarriers of Step 2 at the S-band. (**a**) Using the paper cup; (**b**) Using the plastic bottle; (**c**) Using the glass bottle.

In the S-band environment, the WCI amplitude ranges of the three kinds of suspected liquids differed greatly, as shown in Figure 8.

**Figure 9:**
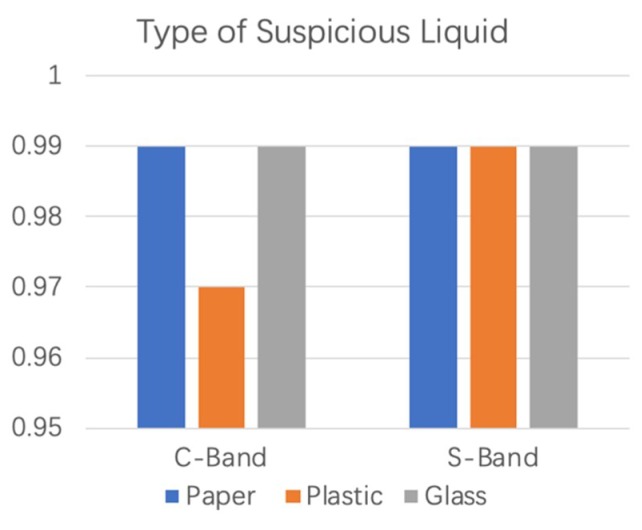
The KNN algorithm Classification results of Step 2 at the C-band and the S-band.

Figure 9 is the result of the KNN classification algorithm in identifying the types of suspicious liquids. In the C-band environment, the system can achieve more than 97% accuracy in identifying types of suspicious liquid (Step 2), and that for the S-band environment is 99%.

### 4.3. Detection of Different Concentrations of Alcohol

The dielectric constant of different liquids was quite different. Even for the same liquid, different concentrations had a certain effect on the dielectric constant. To verify this, we selected 50% alcohol, 75% alcohol, and 95% alcohol to carry out the experiment. Due to the different physical materials of the container, the WCI amplitude of each subcarrier was also affected by different containers in the same band environment. In addition, the amplitude of the WCI varied with the same kind of container at different wavelengths.

**Figure 10:**
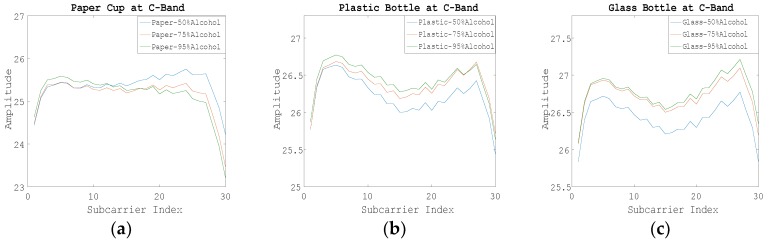
The wireless channel information (WCI) amplitudes of 30 subcarriers corresponding to different concentrations of alcohol at the C-band by using different containers. (**a**) Using the paper cup; (**b**) Using the plastic bottle; (**c**) Using the glass bottle.

Figure 10 shows the WCI amplitudes of 30 subcarriers corresponding to different concentrations of alcohol in the C-band where the paper cup, plastic bottle, and glass bottle were detected. We can see that when the object is in the C-band environment, various containers performed differently when identifying different concentrations of alcohol, but they still accurately identified different concentrations of alcohol. This experiment verifies that the types of containers will not affect the system’s identification of liquids, and further illustrates the reliability of our system in detecting and identifying different liquids. The conclusion is also applicable to the experimental measurement in the S-band environment.

**Figure 11:**
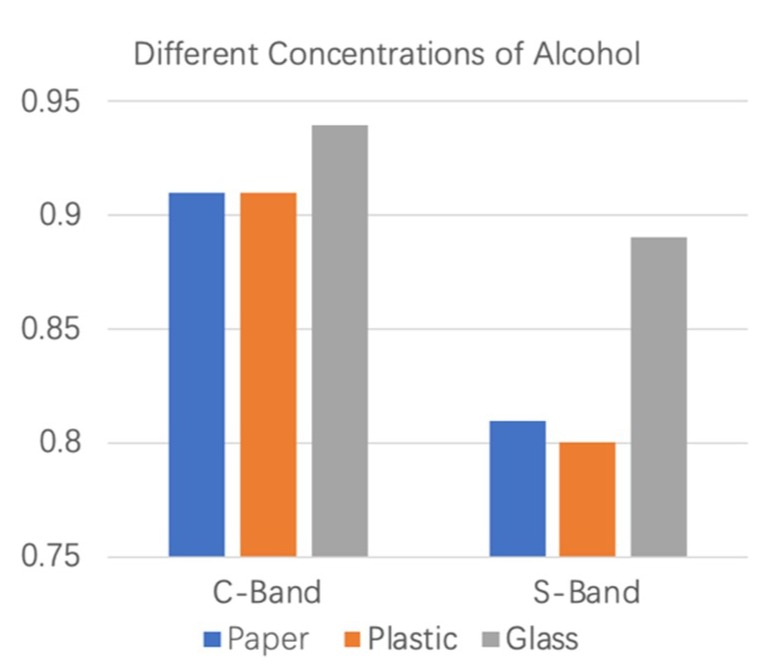
Detection results of different concentrations of alcohol at the C-band and the S-band.

Figure 11 shows the detection results of different concentrations of alcohol in C-band and S-band environments. From the figure, we can see that the detection accuracy of the C-band is higher than that of the S-band for the detection of alcohol with different concentrations. In the C-band frequency analysis experiment, the accuracy of the system for the detection of different concentrations of alcohol reached more than 91%, and that of S-band electromagnetic waves was up to 89%. Therefore, the C-band electromagnetic wave is superior to the S-band electromagnetic wave in the accurate detection of different concentrations of the same liquid.

As can be seen from Table 2, wireless sensing corresponding the 5G frequency band had excellent detection results for the detection and identification of suspicious liquids, no matter which container was selected. Moreover, our method had better performance and robustness in detecting different concentrations of alcohol, and had more subtle differences in dielectric constant than existing Wi-Fi technologies.

## 5. Conclusions

In this work, we explored the feasibility of using a wireless signal in a multi-band environment to detect suspicious liquids. Our work is novel because it demonstrates that it is possible to detect suspicious liquids accurately using radio signals without installing expensive liquid detection machines. Our system can not only detect whether the liquid is suspicious, but also further identify the types of suspicious liquids. In addition, we confirmed the feasibility of liquid concentration detection by using the WCI at a 5G frequency band.

The results analysis shows that our method can accurately detect suspicious and nonsuspicious liquids (Experiment 1) with more than 98% accuracy, regardless of the type and size of containers, and can identify the type of suspicious liquids (Experiment 2) with more than 97% accuracy. For the detection of alcohol with different concentrations, the accuracy can reach up to 94%. This provides better performance and robustness than existing Wi-Fi technology.

However, our method has limitations for liquids stored in metal containers. In that case, we recommend that security personnel intervene. Our method could be further improved by increasing the number of suspicious liquids prohibited in public and looking for ways to reduce the impact of metal containers on liquid detection.

## Figures and Tables

**Table 1:** The relative dielectric constants of common objects.

Object	Dielectric Constant
Water	80
Alcohol	24
Oil	2
Glycerol	37
Methanol	32
Sulfuric Acid	84

**Table 2:** The KNN algorithm classification results of the system.

Band Selection	C-Band			S-Band		
Container	Paper	Plastic	Glass	Paper	Plastic	Glass
Experiment 1	0.98	0.99	0.98	0.99	0.99	0.99
Experiment 2	0.99	0.97	0.99	0.99	0.99	0.99
Experiment 3	0.91	0.91	0.94	0.81	0.80	0.89
